# Effects of exercise interventions on subjective sleep quality in older adults: a systematic review and meta-analysis of studies using the Pittsburgh sleep quality index

**DOI:** 10.3389/fmed.2025.1664567

**Published:** 2025-10-02

**Authors:** Yafan Li, Wei Quan, Ziqi Gao, Xinyi Wang, Yaxin Wang, Hongnan Meng, Jianxin Kang

**Affiliations:** Postgraduate School, Harbin Sport University, Harbin, China

**Keywords:** physical exercise, sleep, older adults, subgroup analysis, meta-analysis

## Abstract

**Objective:**

In accordance with the 2024 Physical Activity Guidelines, this study aimed to evaluate the effects of exercise interventions on subjective sleep quality in older adults and to explore the potential dose–response relationship.

**Methods:**

A systematic search was conducted in Web of Science, PubMed, Cochrane Library, Scopus, and Embase for randomized controlled trials (RCTs) published up to May 1, 2025. Meta-analysis was performed using R, with standardized mean differences (SMD) and 95% confidence intervals (95% CI) used to quantify effect sizes. Subgroup analysis, meta-regression, and nonlinear dose–response modeling were conducted.

**Results:**

A total of 26 RCTs involving 2,189 elderly participants were included. The meta-analysis revealed that exercise interventions significantly improved subjective sleep quality [SMD = −2.46, 95% CI (−2.99, −1.93), *p* < 0.001]. The most pronounced effects were observed in interventions with session durations ≤30 min [WMD = −4.25, 95% CI (−5.49, −3.02)], low intensity [WMD = −2.79, 95% CI (−3.44, −2.14)], twice-weekly frequency [WMD = −2.52, 95% CI (−3.00, −2.04)], and intervention durations ≤8 weeks [WMD = −2.45, 95% CI (−2.99, −1.91)]. Meta-regression showed no significant linear associations between sleep outcomes and intervention duration, intensity, frequency, or length. A nonlinear “U-shaped” dose–response relationship was identified, with the optimal effect observed at approximately 527 MET·min/week [Hedges’ g = −0.82, 95% CI (−1.12, −0.52)].

**Conclusion:**

Low-frequency, short-duration, and low-to-moderate intensity exercise interventions can effectively improve subjective sleep quality in older adults. Notably, even low-dose exercise can yield significant benefits.

## Introduction

With the global trend of population aging intensifying, the growing burden of the elderly population on healthcare systems and socioeconomic development has made age-related diseases a pressing public health concern (1). Studies have shown that older adults frequently suffer from a range of chronic health conditions, among which sleep disturbances are among the most common, significantly impairing both physical and mental health as well as quality of life (2, 3). Compared to younger adults, older individuals are more prone to disruptions in sleep architecture, reductions in deep sleep, and disturbances in circadian rhythms, often manifested as difficulty falling asleep, early morning awakening, and frequent nocturnal awakenings (4).

Sleep disturbances are not only considered early indicators of frailty syndrome in older adults but are also closely associated with adverse outcomes such as hypertension, coronary heart disease, cognitive decline, depression, and increased mortality (5, 6). Although pharmacological interventions remain the primary approach to managing sleep disorders, the long-term use of hypnotic medications may lead to drug dependence, cognitive impairment, and a heightened risk of falls, rendering such treatments inadequate for meeting the needs of safe, sustainable, and effective rehabilitation in elderly populations (7). Therefore, the development of non-pharmacological interventions—particularly those involving safe and sustainable lifestyle modifications—is of critical importance for improving sleep health in older adults.

Growing evidence suggests that exercise, as a planned and repetitive form of physical activity, can effectively mitigate age-related health risks by enhancing muscular strength, improving cardiopulmonary function, and regulating metabolic and neuroendocrine processes (8). In the context of sleep, exercise interventions have been shown to improve subjective sleep quality, reduce sleep onset latency, and decrease nighttime awakenings, while also potentially reducing reliance on pharmacological treatments (2, 9, 10). However, there remains a lack of consensus regarding the optimal type, intensity, frequency, and duration of exercise for improving sleep, particularly among older adults. The formulation of safe, evidence-based, and personalized intervention strategies remains underexplored in this population. The Pittsburgh Sleep Quality Index (PSQI) was chosen as the primary outcome because it is widely validated in older adults, sensitive to intervention effects, and provides a multidimensional assessment of sleep quality beyond single indicators (11, 12).

To address this gap, the present study employed a systematic review and meta-analytic approach to synthesize existing randomized controlled trial (RCT) data, with the aim of evaluating the effects of exercise interventions on subjective sleep quality in older adults. Furthermore, the dose–response relationship between intervention parameters and sleep outcomes was investigated, in order to provide both theoretical and practical guidance for developing individualized exercise prescriptions tailored to the physiological characteristics of the elderly (13).

## Methods

This systematic review was conducted in strict accordance with the Preferred Reporting Items for Systematic Reviews and Meta-Analyses (PRISMA) guidelines (14) and was prospectively registered in the PROSPERO database (Registration No. CRD42025636756).

### Search strategy

A comprehensive literature search was performed across five electronic databases: PubMed, Web of Science, Scopus, Embase, and the Cochrane Library, covering all records from database inception to May 1, 2025. The search strategy for PubMed was as follows: (“sleep” [Title/Abstract] OR “sleep quality” [Title/Abstract] OR “slumber” [Title/Abstract] OR “rest” [Title/Abstract]) AND (“exercise” [Title/Abstract] OR “physical exercise” [Title/Abstract] OR “exercise training” [Title/Abstract] OR “physical activity” [Title/Abstract] OR “workout” [Title/Abstract]) AND (“older adults” [Title/Abstract] OR “elderly” [Title/Abstract] OR “aged adults” [Title/Abstract] OR “seniors” [Title/Abstract] OR “middle-aged and older adults” [Title/Abstract]) AND (randomized controlled trial [Filter]).

The detailed search strategy for each database is presented in Supplementary Appendix A. The literature screening process was conducted independently and in duplicate by two reviewers using a blinded method. Any disagreements were resolved by consultation with a third reviewer.

### Inclusion criteria

1. Language: Only studies published in English were considered;
2. Participants: Studies involving older adults aged 60 years and above without medical conditions that severely impair sleep were included;
3. Study Design: Only randomized controlled trials (RCTs) were included;
4. Intervention: The experimental group received regular exercise interventions over a defined period, while the control group received no exercise intervention;
5. Type of Intervention: Various exercise modalities were accepted, including differences in content, intensity, duration, frequency, and intervention period.

### Exclusion criteria

1. Review articles, conference abstracts, or case studies;
2. Animal studies;
3. Studies involving only a single exercise session rather than repeated or long-term interventions;
4. Duplicated publications, studies of poor methodological quality, or those for which the full text could not be obtained;
5. Studies that did not report the Pittsburgh Sleep Quality Index (PSQI) as an outcome measure.

### Study selection and data extraction

Two reviewers independently screened the literature based on pre-specified inclusion and exclusion criteria. Initially, titles and abstracts were reviewed to exclude studies irrelevant to the research topic. Full-text screening was subsequently conducted for potentially eligible studies to determine their final inclusion. Any discrepancies during the selection process were resolved through discussion with a third reviewer.

Data extraction was conducted concurrently with the full-text review. The extracted information included the first author, year of publication, study location, study design, sample size, participant age, type of exercise, exercise intensity, frequency, duration per session, intervention period, and the primary outcome measure (Pittsburgh Sleep Quality Index, PSQI). In all included studies, the PSQI was assessed both at baseline and post-intervention to evaluate changes in subjective sleep quality. For the purpose of this meta-analysis, subjective sleep quality was operationalized as the global PSQI score, unless otherwise specified. Only the global score was used for analysis; individual PSQI components (e.g., total sleep time, sleep latency) were not examined separately due to inconsistent reporting across studies.

For multiple publications originating from the same trial, only one version was included to avoid duplicate data. The publication that best matched the research objectives and provided the most complete data—typically the most recent version—was prioritized, while others were excluded. To minimize subjectivity in the classification of exercise variables, the following criteria were applied:

1. Fixed values of intensity, frequency, and duration were recorded when explicitly reported;
2. For variables presented as ranges, the average value was calculated;
3. Exercise intensity, when reported using different scales (e.g., Borg 15-point scale or Borg 10-point scale), was standardized using the Borg CR10 scale for dose–response analysis (15). Specifically, the studies by Chen et al. (16); Chen et al. (17); and Siu et al. (18) reported the intensity directly using the CR10 scale; the studies by King (19); Sharif et al. (20); and Sternfeld et al. (21) used the Borg RPE15 scale and were therefore converted to CR10; all remaining studies reported exercise intensity as a percentage of maximum heart rate or oxygen uptake and were converted using percentage-based approximations.

### Quality assessment

The risk of bias and methodological quality of included RCTs were assessed using the Cochrane Risk of Bias (RoB) tool. This tool evaluates seven domains: random sequence generation, allocation concealment, blinding of participants and personnel, blinding of outcome assessors, incomplete outcome data, selective reporting, and other potential sources of bias.

Each domain was rated as “low risk,” “unclear risk,” or “high risk” of bias. The assessment was independently conducted by two reviewers based on the original reports. In cases of disagreement, a third reviewer participated in discussion to reach consensus, thereby ensuring objectivity and consistency in the evaluation process.

### Statistical analysis

Meta-analyses were performed using R software. Subgroup analyses were conducted to examine the effects of different intervention characteristics. Heterogeneity was assessed using Cochran’s Q test, with a significance level set at *α* = 0.1. The degree of heterogeneity was quantified using the *I^2^* statistic: *I^2^* < 50% and *p* > 0.1 were interpreted as low heterogeneity, in which case a fixed-effects model was applied; *I^2^* ≥ 50% and *p* < 0.1 indicated substantial heterogeneity, warranting the use of a random-effects model.

The primary outcome was treated as a continuous variable. For studies employing the same sleep quality scale, weighted mean differences (WMD) with 95% confidence intervals (CI) were calculated. For studies using different scales, standardized mean differences (SMD) with 95% CI were computed and used in sensitivity analyses.

To explore the relationship between intervention dose and sleep improvement, a nonlinear dose–response meta-analysis was conducted. A random-effects one-stage mixed-effects model was employed to simultaneously estimate the dose–response relationship across all studies. Restricted cubic spline models and maximum likelihood estimation were applied at fixed percentiles (5, 50, and 95%) to model the relationship between exercise dose (expressed in MET-minutes/week) and improvements in subjective sleep quality, without assuming a predefined functional form. Exercise dose was calculated as the product of session duration, frequency, and intensity. A reference dose of zero was used for estimating relative effect sizes under varying exercise exposure conditions.

## Results

### Search results

A systematic search of five databases (PubMed, Web of Science, Cochrane Library, Scopus, and Embase) yielded a total of 1,899 relevant records. After removing 642 duplicates, 1,257 studies remained for title and abstract screening. During this stage, 112 articles were excluded, including 6 non-English publications, 74 non-randomized controlled trials, and 32 with incomplete data. A total of 1,145 articles were retrieved for full-text review, of which 56 could not be obtained. Among the 1,089 reports assessed for eligibility, 1,046 were excluded for the following reasons: outcome indicators not involving the Pittsburgh Sleep Quality Index (PSQI; *n* = 267), study participants not meeting inclusion criteria (*n* = 340), inappropriate form of intervention in the control group (*n* = 95), disease type not in line with inclusion criteria (*n* = 317), meta-analysis articles (*n* = 23), and animal experiments (*n* = 4).

**Figure 1:**
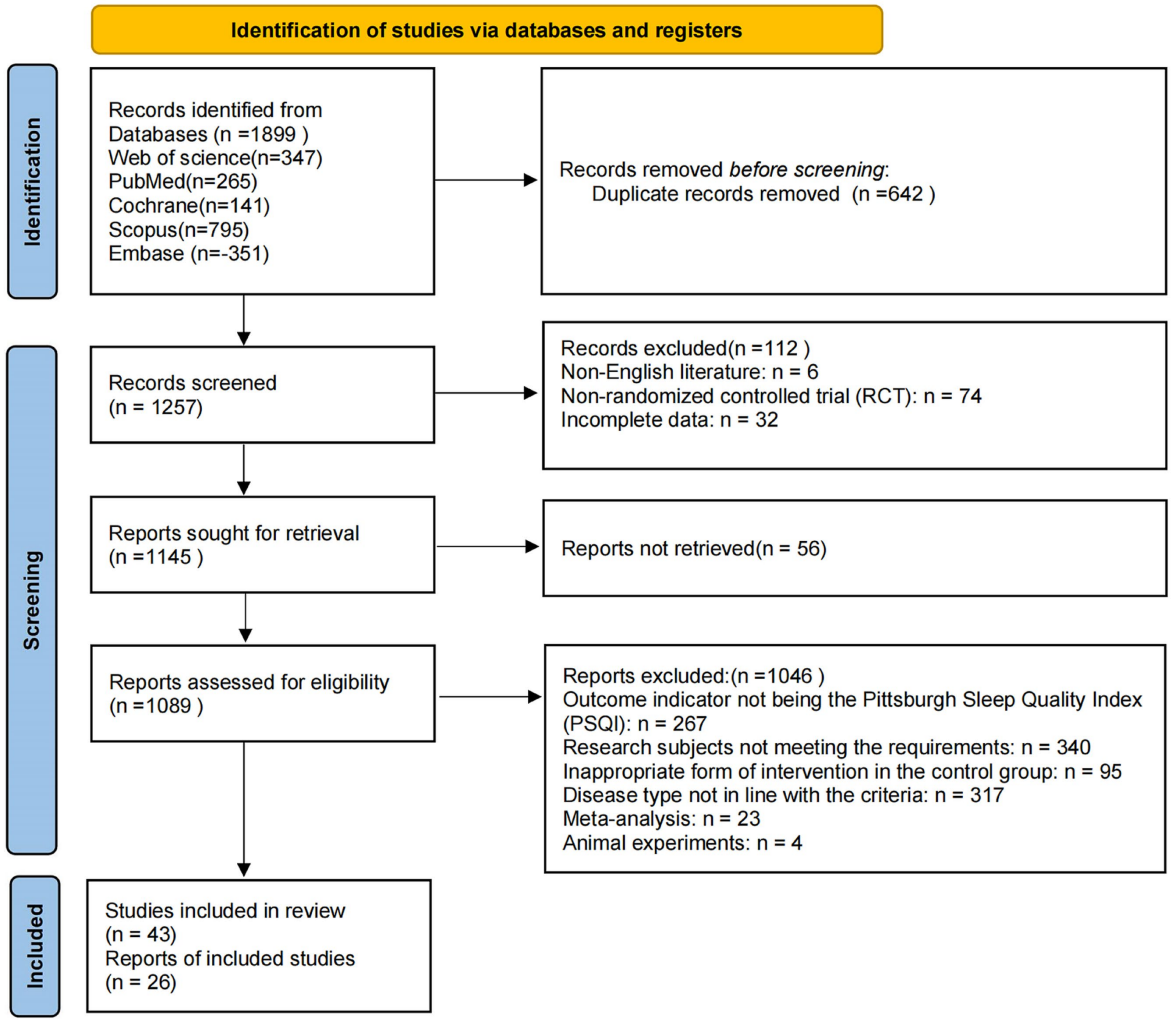
PRISMA flow diagram of literature selection.

Following full-text assessment, 26 randomized controlled trials were identified as eligible and included in the systematic review and meta-analysis (17–42). The study selection process is illustrated in Figure 1. In this study, “subjective sleep quality” was consistently defined as the global PSQI score, unless otherwise specified. Only the global score of the PSQI was used for the meta-analysis, and individual components such as total sleep time or sleep latency were not analyzed separately due to inconsistent reporting across studies.

### Characteristics of included studies

A total of 26 randomized controlled trials (17–42) involving a total of 2,189 older adults aged between 60 and 90 years were included in the analysis.

In terms of exercise type, several studies employed multicomponent programs integrating aerobic, resistance, flexibility, and balance training (19, 22–24, 30, 37, 41), while the remaining trials adopted single-modality interventions, such as: Tai Chi: (20, 26, 29); Yoga, Pilates, or stretching: (17, 34); Aerobic walking or dance: (21, 27, 35, 36); Progressive resistance training: (31, 38).

With respect to exercise intensity, most multicomponent programs and aerobic walking interventions were conducted at a moderate intensity; Tai Chi, yoga, and stretching were generally considered low-intensity mind–body exercises; whereas Singh et al. (38) and Karimi et al. (31) implemented high-intensity progressive resistance training protocols.

Regarding intervention duration, the included studies ranged from 6 weeks to 12 months, with the majority lasting 12 to 24 weeks. Training frequency was typically set at 2–5 sessions per week across studies.

Overall, the included trials encompassed exercise modes ranging from low to high intensity and intervention durations from short to long term. Detailed information on exercise types, intensity, frequency, and duration is presented in Table 1.

**Table 1:** Characteristics of included studies.

First Author	Country	Sample size EG CG		Age EG CG		Intervention EG CG		Frequency and duration	Session time/min	Intensity	Main outcome
Mahajan et al. (34)	India	6	7	74.16 ± 6.17	71.33 ± 6.25	Interval training	Sleep Hygiene Education(SH)lecture	3times 8 weeks	30-40 min	Low to high intensity	PSQI
Alcalá et al. (2024)	Spain	47	45	71.43 ± 2.97	72.24 ± 2.92	Dance-based aerobic training	Daily activities with encouragement to maintain them	2times 12 weeks	60 min	Moderate intensity	PSQI
King (19)	USA	20	23	62.4 ± 6.4	61.2 ± 7.5	Low-impact aerobic exercise and walking	Chrononutrition intervention	4times 16 weeks	60 min	Moderate intensity	PSQI
Aibar-Almazán et al. (22)	Spain	55	55	69.98 ± 7.83	66.79 ± 10.14	Pilates,stretching,and mat training	Guideline-based daily habits with no additional exercise	2times 12 weeks	60 min	Low to moderate intensity	PSQI
Baklouti et al. (23)	Tunisia	65	95	65 ± 8.5	65 ± 8.5	Hatha yoga training	No intervention	2times 8 weeks	80 min	Low to moderate intensity	PSQI
Carcelén-Fraile et al. (24)	Spain	53	55	69.73 ± 2.56	70.45 ± 2.68	Resistance training	Maintaining daily activities	2times 12 weeks	60 min	Moderate intensity	PSQI
Chen et al. (25)	Taiwan	62	66	65.77 ± 4.32	72.42 ± 6.04	Silver yoga program	Routine activities at elderly centers	3times 6 months	70 min	Low intensity	PSQI
Chen et al. (17)	Taiwan	38	31	75.4 ± 6.7	75.4 ± 6.7	Silver yoga exercises	Routine activities at institutions	3times 6 months	70 min	Low intensity	PSQI
Chen et al. (26)	Taiwan	27	28	71.75 ± 8.13	71.75 ± 8.14	Baduanjin Qigong exercise	Routine activities at institutions	3times 12 weeks	30 min	Moderate intensity	PSQI
Choi (35)	Korea	33	30	77.6 ± 5.69	78.8 ± 5.83	Floor-sitting exercise program (FSEP)	Routine care	4times 12 weeks	30-40 min	Low to moderate intensity	PSQI
Curi et al. (27)	Brazil	31	30	64.25 ± 0.14	63.75 ± 0.08	Pilates training	No physical activities	2times 16 weeks	60 min	Low to moderate intensity	PSQI
Frye et al. (28)	USA	23	21	69.2 ± 9.26	69.2 ± 9.27	Tai Chi	No exercise intervention	3times 12 weeks	60 min	Low intensity	PSQI
Li et al. (29)	USA	43	32	75.30 ± 7.8	75.45 ± 7.8	Tai Chi training	Low-impact exercise	3times 24 weeks	60 min	Low to moderate intensity	PSQI
Jimenez-Garcia et al. (30)	Spain	26	23	68.23 ± 2.97	68.52 ± 6.33	Suspension training HIIT	Maintaining daily life	2times 12 weeks	45 min	High intensity	PSQI
Karimi et al. (31)	Iran	23	23	67.49 ± 4.28	66.82 ± 3.84	Walking plan,specific activities	No intervention	3times 8 weeks	30 min	Low intensity	PSQI
King et al. (33)	USA	32	27	61.86 ± 6.33	60.90 ± 7.19	Moderate-intensity endurance exercise	health education training	5times 12 months	60 min	Moderate to high intensity	PSQI
Kerkez and Erci (32)	Turkey	58	56	70.89 ± 4.23	71.80 ± 4.03	Tai Chi Qigong	No intervention	3times 6 weeks	35-40 min	Low intensity	PSQI
Nguyen and Kruse (36)	Vietnam	39	34	69.2 ± 5.3	68.7 ± 4.9	Tai Chi training	Maintaining daily activities	2times 6 months	60 min	Low intensity	PSQI
Şekerci and B. (42)	Turkey	23	30	73.56 ± 8.25	71.91 ± 7.98	Walking plan	No intervention	2times 8 weeks	40 min	Moderate intensity	PSQI
Sharif et al. (20)	Iran	23	30	64.8 ± 5.2	64.8 ± 5.3	Aerobic exercise program	No intervention	3times 12 weeks	60 min	Moderate intensity	PSQI
Singh et al. (38)	USA	15	13	70.0 ± 1.6	72.0 ± 1.9	Progressive resistance training	Health education program	3times 10 weeks	60 min	High intensity	PSQI
Siu et al. (18)	Hong Kong	105	110	66.5 ± 6.4	68.0 ± 8.2	Tai Chi	No intervention	3times 12 weeks	60 min	Low to moderate intensity	PSQI
Song et al. (40)	China	18	17	64.15 ± 8.56	64.15 ± 8.57	Modified Tai Chi training	home exercise guidance	3times 12 weeks	60 min	Low to moderate intensity	PSQI
Song et al. (39)	China	36	35	76.71 ± 5.96	75.20 ± 6.63	Aerobic dance program	health education and home exercise guidance	3times 16 weeks	60 min	Moderate intensity	PSQI
Sternfeld et al. (21)	USA	106	142	55.8 ± 3.6	54.2 ± 3.5	Aerobic exercise	No changes to physical activity behavior	3times 12 weeks	40-60 min	Moderate to high intensity	PSQI
Wang et al. (41)	China	30	25	66.0 ± 4.97	69.1 ± 4.56	Tai Chi practice	Online health lecture	3times 8 weeks	60 min	Low to moderate intensity	PSQI

### Quality assessment results

All 26 included studies (17–42) were evaluated using the Cochrane Risk of Bias assessment tool.

**Figure 2:**
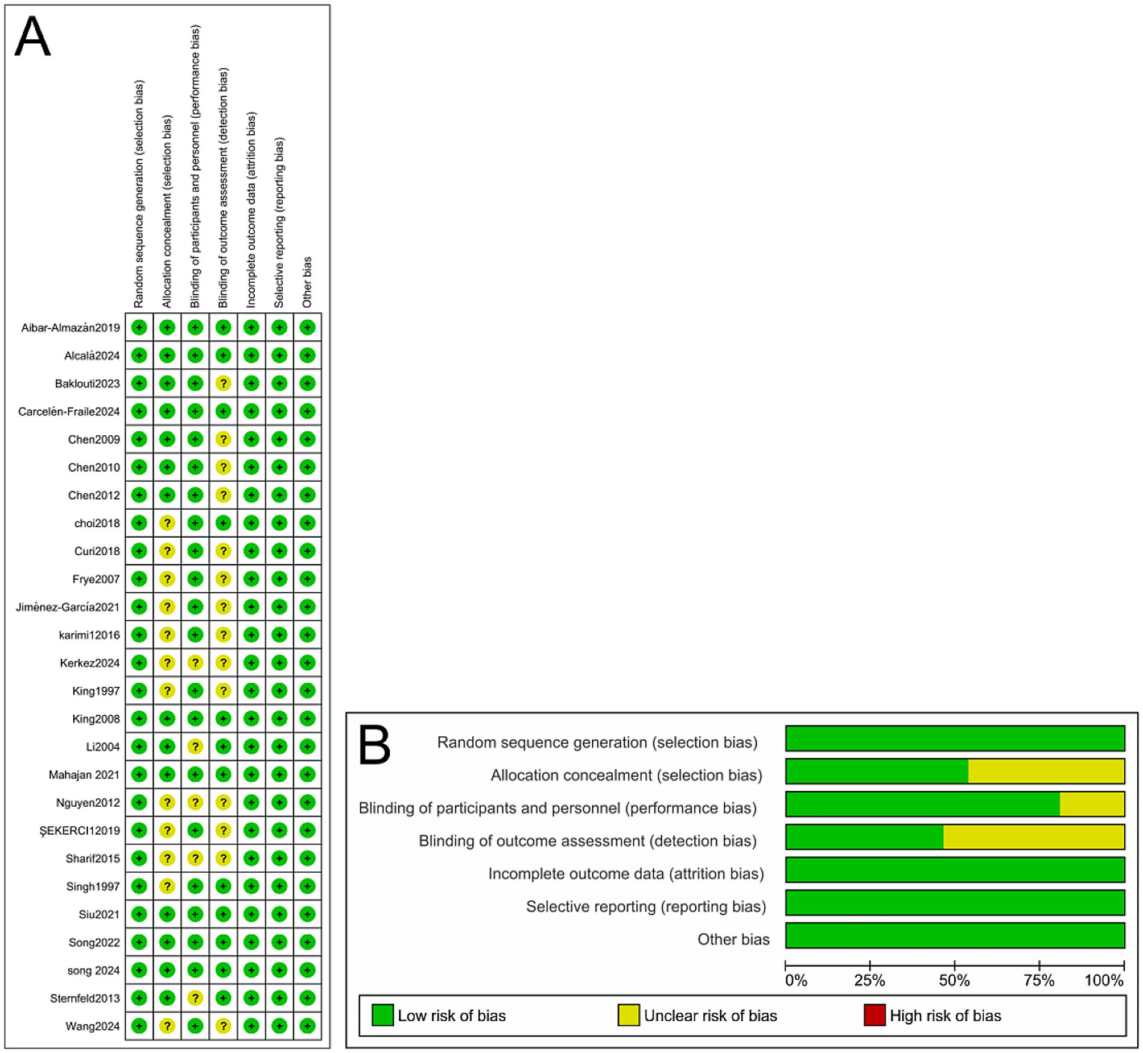
**(A)** Methodological quality assessment of 26 included RCTs. **(B)** Risk-of-bias summary and risk-of-bias graph for 26 RCTs.

The results indicated that most studies exhibited a low risk of bias in domains such as random sequence generation, blinding of outcome assessors, and handling of incomplete outcome data, suggesting a generally high methodological quality. However, some studies provided unclear or insufficient descriptions regarding the blinding of participants and personnel, indicating a potential risk of performance bias in these domains. Detailed results of the quality assessment are presented in Figure 2.

### Meta-analysis results

#### Effect of exercise on sleep in older adults

This meta-analysis included 26 studies (17–42) evaluating the effects of exercise interventions on subjective sleep quality in older adults.

**Figure 3:**
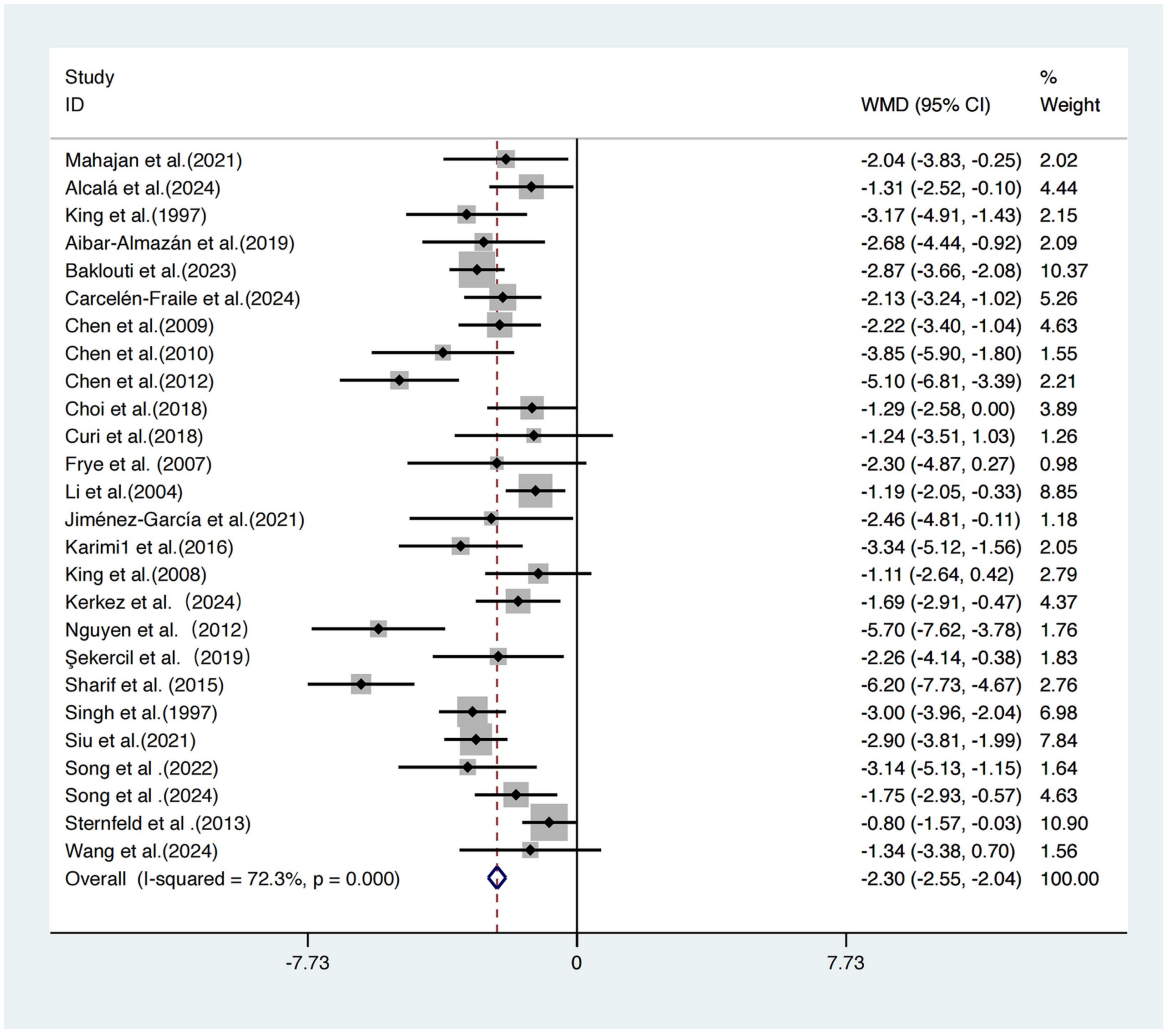
Forest plot of the effect of exercise intervention on sleep quality in older adults.

Due to significant heterogeneity across studies (*I^2^* = 72.3%, τ^2^ = 1.26, *p* < 0.001), a random-effects model was employed. The pooled results showed that exercise interventions significantly improved subjective sleep quality compared to control conditions, with a statistically significant effect size standardized mean difference [SMD = −2.30, 95% CI (−2.55, −2.04), *p* < 0.001] (Figure 3).

### Publication bias

**Figure 4:**
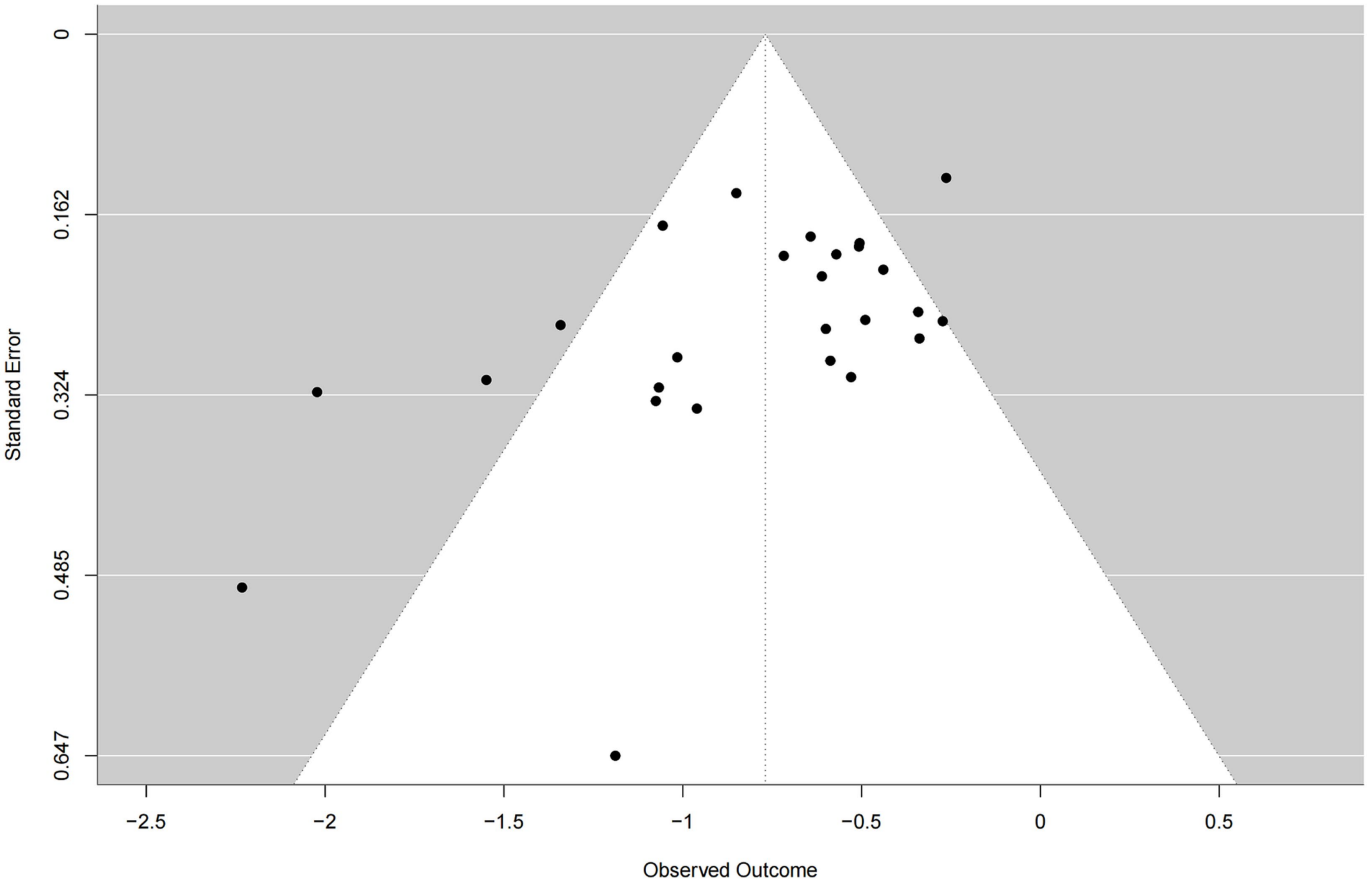
Funnel plot for publication bias in studies assessing the effects of exercise on sleep.

The funnel plot demonstrated an approximately symmetrical distribution of studies around the pooled effect estimate and showed convergence from the bottom to the top, suggesting no substantial evidence of publication bias in this meta-analysis (Figure 4).

### Dose–response relationship between exercise and sleep improvement in older adults

**Figure 5:**
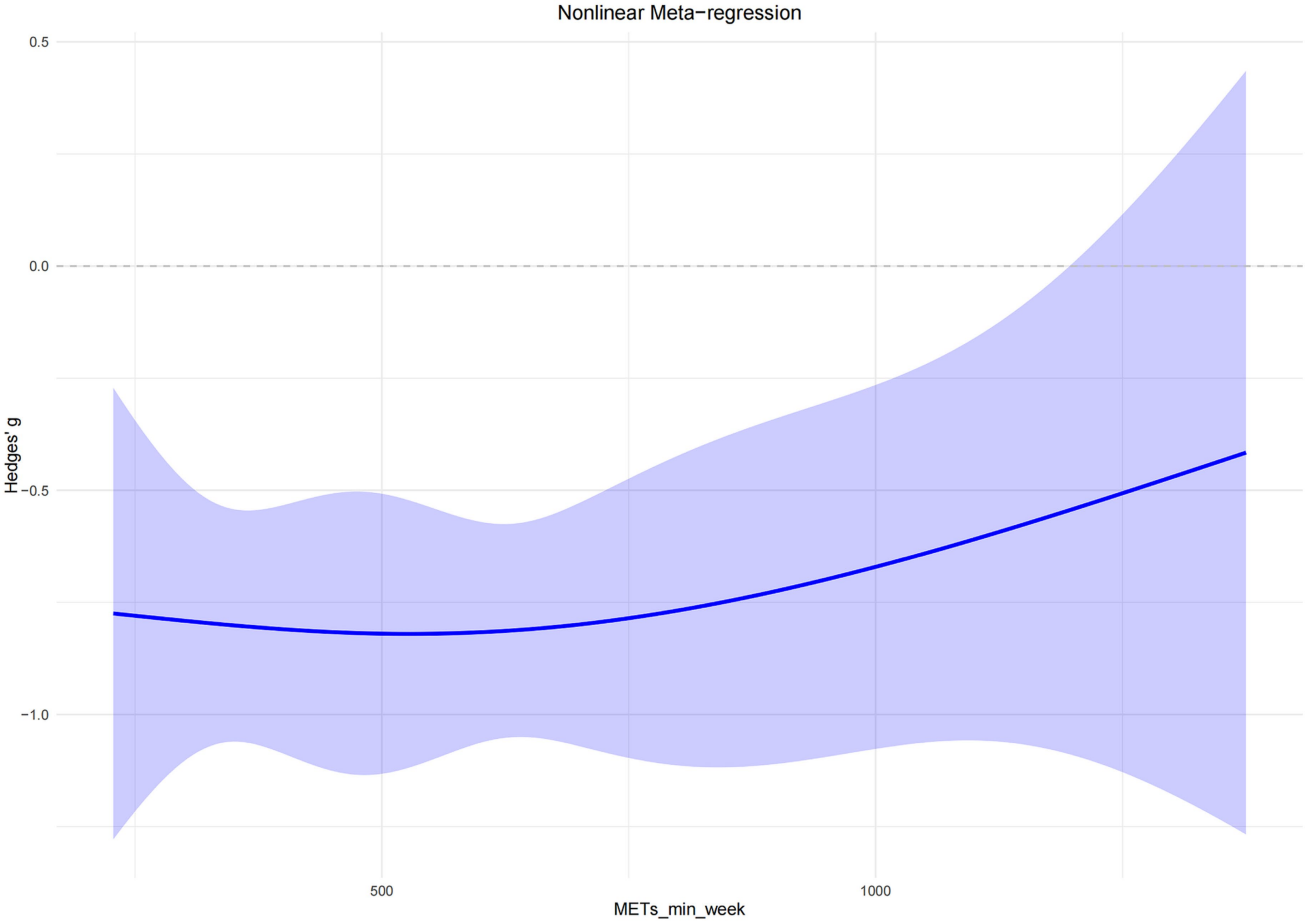
Dose–response relationship between exercise and sleep improvement in older adults.

The overall dose–response curve between exercise dose and sleep improvement in older adults exhibited an inverted U-shaped pattern. In the low-dose range (approximately 200–500 MET-min/week), the intervention effect increased progressively with the amount of exercise. The greatest effect was observed at approximately 527 MET-min/week, representing the lowest point of the curve (Hedges’ g = −0.82), with a predicted effect estimate of −0.82 (95% CI: −1.12, −0.52). As exercise volume continued to increase beyond this point, the effect began to attenuate slightly, as indicated by the upward slope of the curve (Figure 5).

### Subgroup analysis

#### Intervention duration per session

Based on the duration of each exercise session, the 26 included studies (17–42) were categorized into three subgroups for analysis.

The subgroup receiving 0–30-min sessions demonstrated the most pronounced intervention effect, with a weighted mean difference WMDs of −4.25 (95% CI: −5.49, −3.02), and moderate heterogeneity (*I^2^* = 48.8%, *p =* 0.162), indicating relatively stable and consistent results.

The 30–60-min group showed a WMD of −2.09 (95% CI: −2.37, −1.80), but with high heterogeneity (*I^2^* = 72.4%, *p* < 0.001), suggesting greater uncertainty in the results.

The >60-min group reported a WMD of −2.78 (95% CI: −3.41, −2.15) and exhibited the lowest heterogeneity (*I^2^* = 0.0%, *p =* 0.376).

A statistically significant difference was found across subgroups (*p =* 0.001), indicating that session duration is a key moderator of intervention effectiveness.

**Figure 6:**
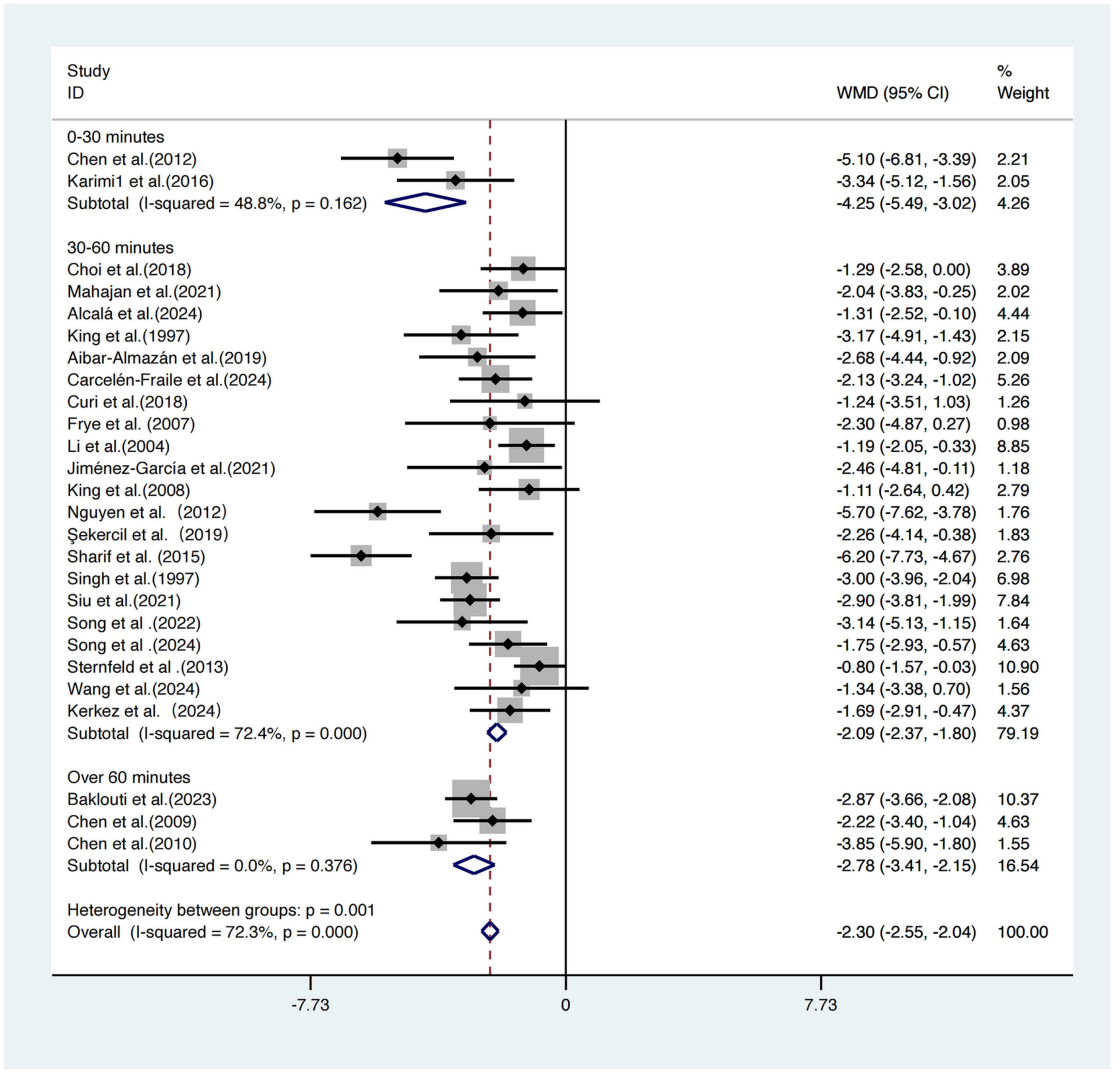
Subgroup analysis of intervention session duration on sleep quality improvement.

Considering the magnitude of the effect size, the width of the confidence intervals, and heterogeneity levels, exercise sessions lasting 0–30 min appear to offer a relatively optimal balance between efficacy and practical feasibility (Figure 6).

### Intervention intensity

A subgroup analysis based on exercise intensity was conducted across the 26 included studies (17–42).

The subgroup with low-intensity interventions demonstrated the greatest effect size [WMD = −2.79, 95% CI (−3.44, −2.14)] with moderate heterogeneity (*I^2^* = 65.3%). The moderate-intensity group yielded a similar effect size [WMD = −2.77, 95% CI (−3.30, −2.24)] but showed high heterogeneity (*I^2^* = 83.6%). The low-to-moderate intensity group reported a WMD of −2.20 (95% CI: −2.61, −1.78) with moderate heterogeneity (*I^2^* = 52.8%). The high-intensity group showed a WMD of −2.75 (95% CI: −3.55, −1.95) with no observed heterogeneity (*I^2^* = 0.0%).

**Figure 7:**
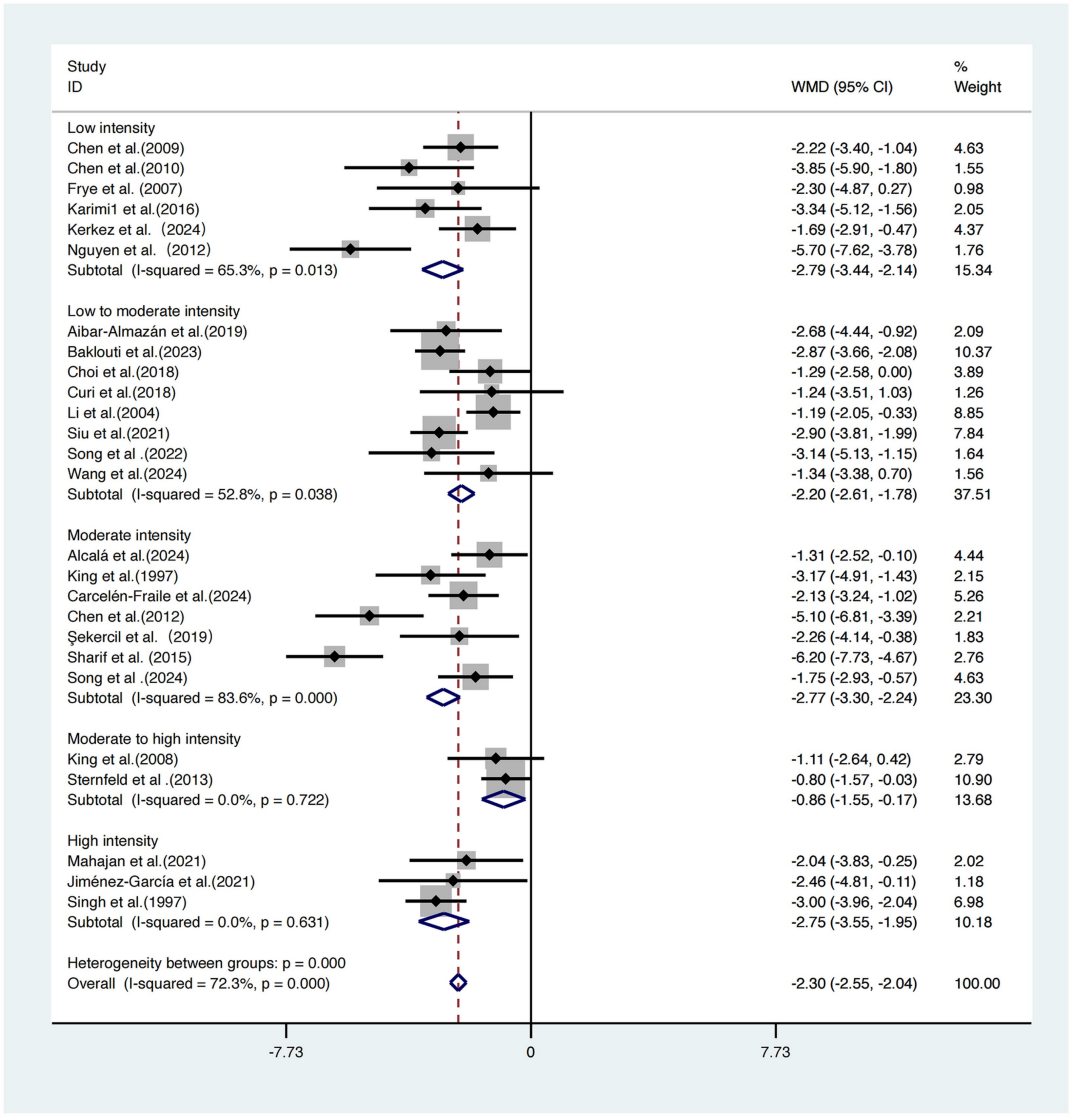
Subgroup analysis of intervention intensity on sleep quality improvement.

By contrast, the moderate-to-high intensity group demonstrated the weakest intervention effect [WMD = −0.86, 95% CI (−1.55, −0.17)]. The differences among subgroups were statistically significant (*p =* 0.000), indicating that intervention intensity plays a critical role in the effectiveness of sleep improvement (Figure 7).

### Intervention frequency

Subgroup analysis based on intervention frequency was performed on the same 26 studies (17–42).

The group exercising twice per week showed the greatest improvement [WMD = −2.52, 95% CI (−3.00, −2.04)]. The group exercising three times per week had a slightly smaller effect size [WMD = −2.28, 95% CI (−2.60, −1.96)]. The group exercising more than three times per week demonstrated the weakest effect [WMD = −1.69, 95% CI (−2.55, −0.83)].

**Figure 8:**
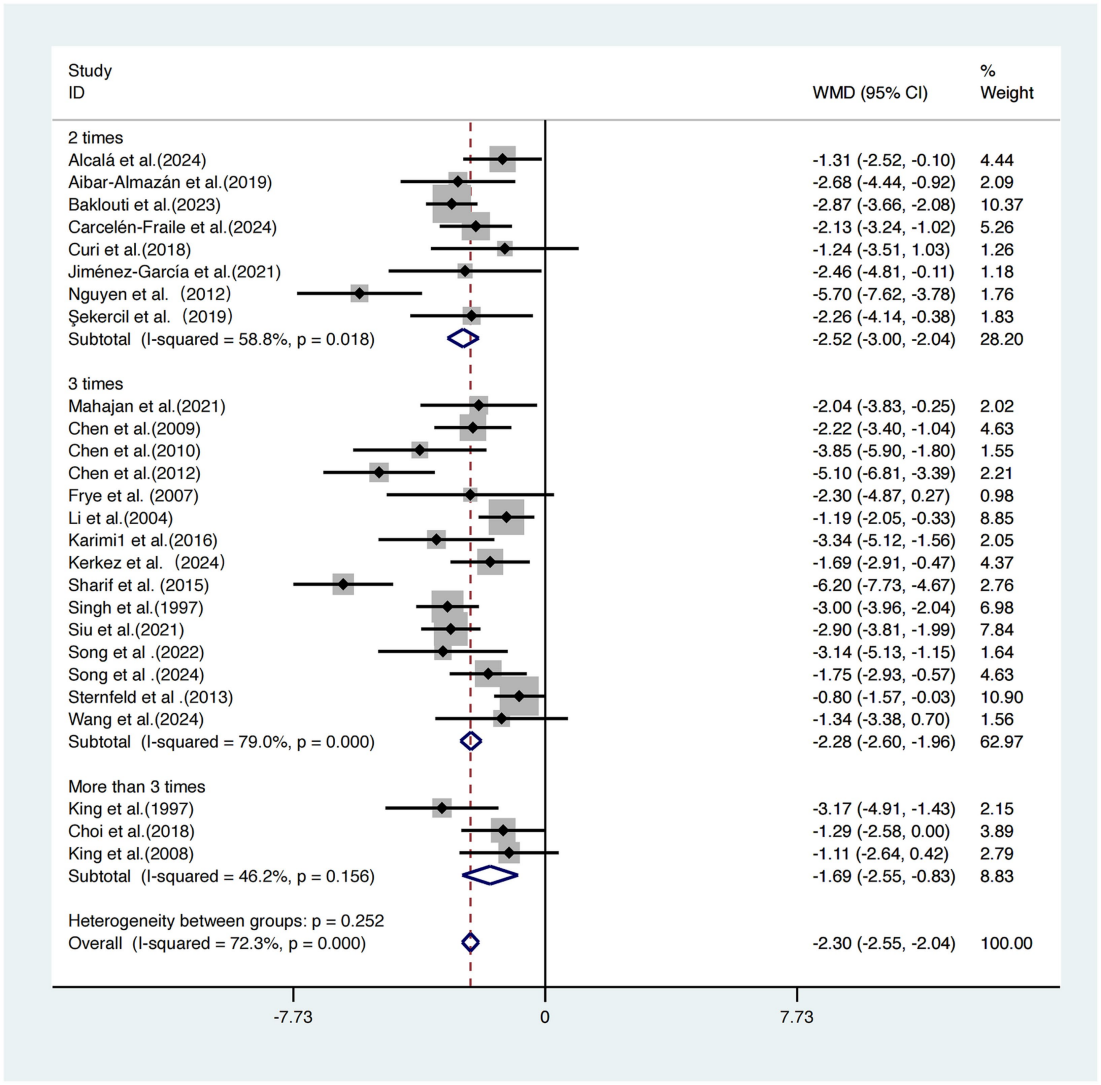
Subgroup analysis of intervention frequency on sleep quality improvement.

Although effect sizes differed among subgroups, the differences were not statistically significant (*p =* 0.252), suggesting that intervention frequency may not be a key determinant of intervention efficacy (Figure 8).

### Intervention duration

The 26 included studies (17–42) were categorized into three groups based on total intervention duration: 0–8 weeks, 8–16 weeks, and >16 weeks.

**Figure 9:**
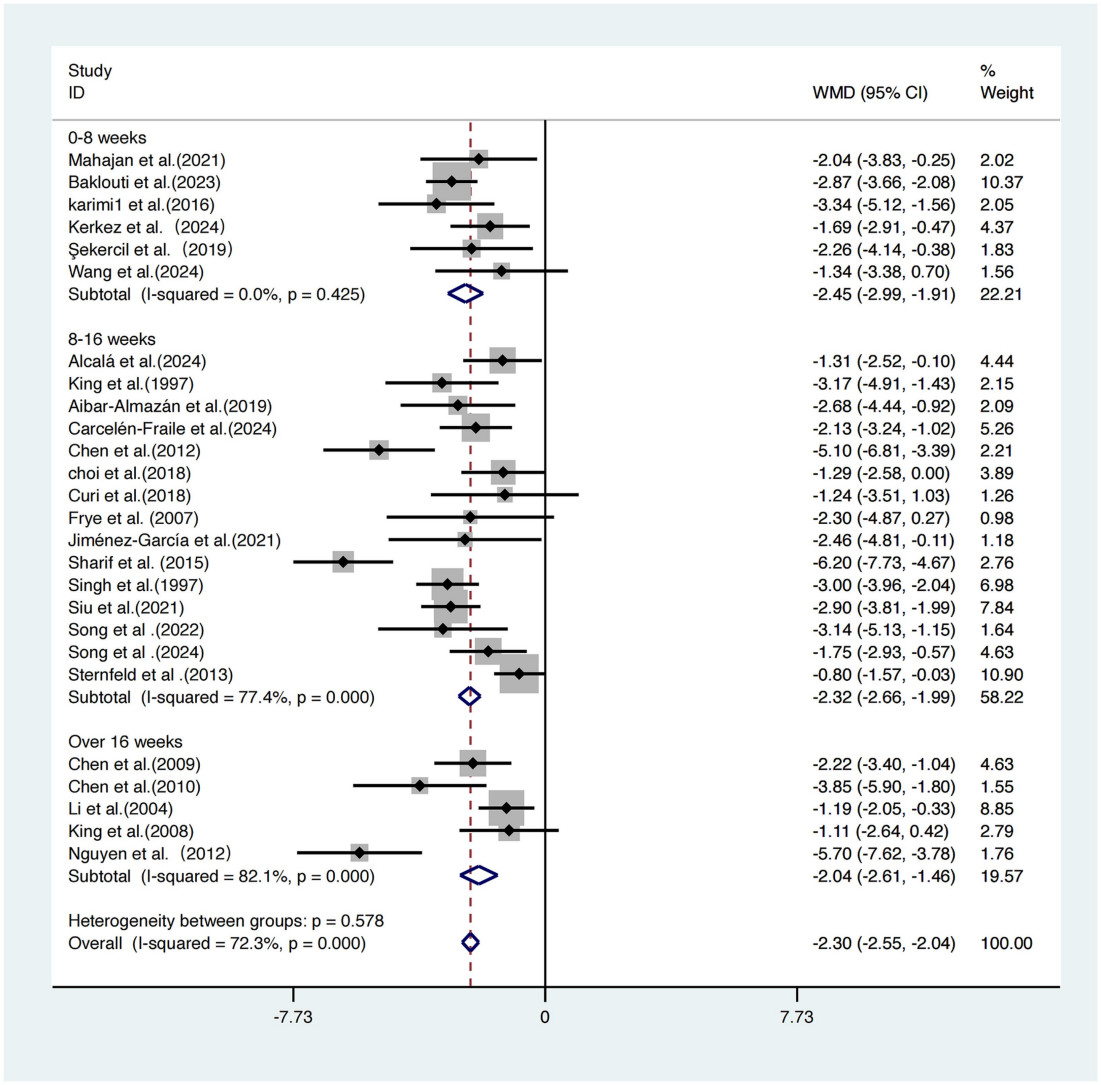
Subgroup analysis of intervention duration on sleep quality improvement.

The short-term intervention group (0–8 weeks) showed the most pronounced improvement in sleep quality [WMD = −2.45, 95% CI (−2.99, −1.91)], with no heterogeneity (*I^2^* = 0.0%), suggesting highly consistent results. The moderate-term (8–16 weeks) and long-term (>16 weeks) groups yielded effect sizes of −2.32 and −2.04, respectively, but both exhibited high heterogeneity (*I^2^* = 77.4 and 82.1%, respectively). Although all three subgroups demonstrated significant improvements in sleep outcomes, the between-group difference was not statistically significant (*p =* 0.578), indicating that total intervention duration may not be a critical factor influencing sleep improvement (Figure 9).

### Intervention modality

A subgroup analysis based on exercise modality was performed using standardized mean differences (SMD) across the same 26 studies. Overall, exercise interventions produced a moderate improvement in sleep among older adults [SMD = −0.79, 95% CI (−0.96, −0.63), *p* < 0.0001], with moderate heterogeneity (*I^2^* = 60.9%).

Among intervention types, dance-based programs showed the strongest effect (SMD = −1.59), albeit with substantial heterogeneity (*I^2^* = 73.5%). Aerobic exercise also yielded a large effect (SMD = −1.03) with high heterogeneity (*I^2^* = 80.7%). In contrast, yoga and tai chi interventions demonstrated stable and consistent effects, with effect sizes of −0.54 and −0.78, respectively, and no heterogeneity (*I^2^* = 0.0%). Resistance training showed a modest effect (SMD = −0.65), but the confidence interval crossed zero, rendering the result statistically non-significant and heterogeneity relatively high (*I^2^* = 73.5%).

**Figure 10:**
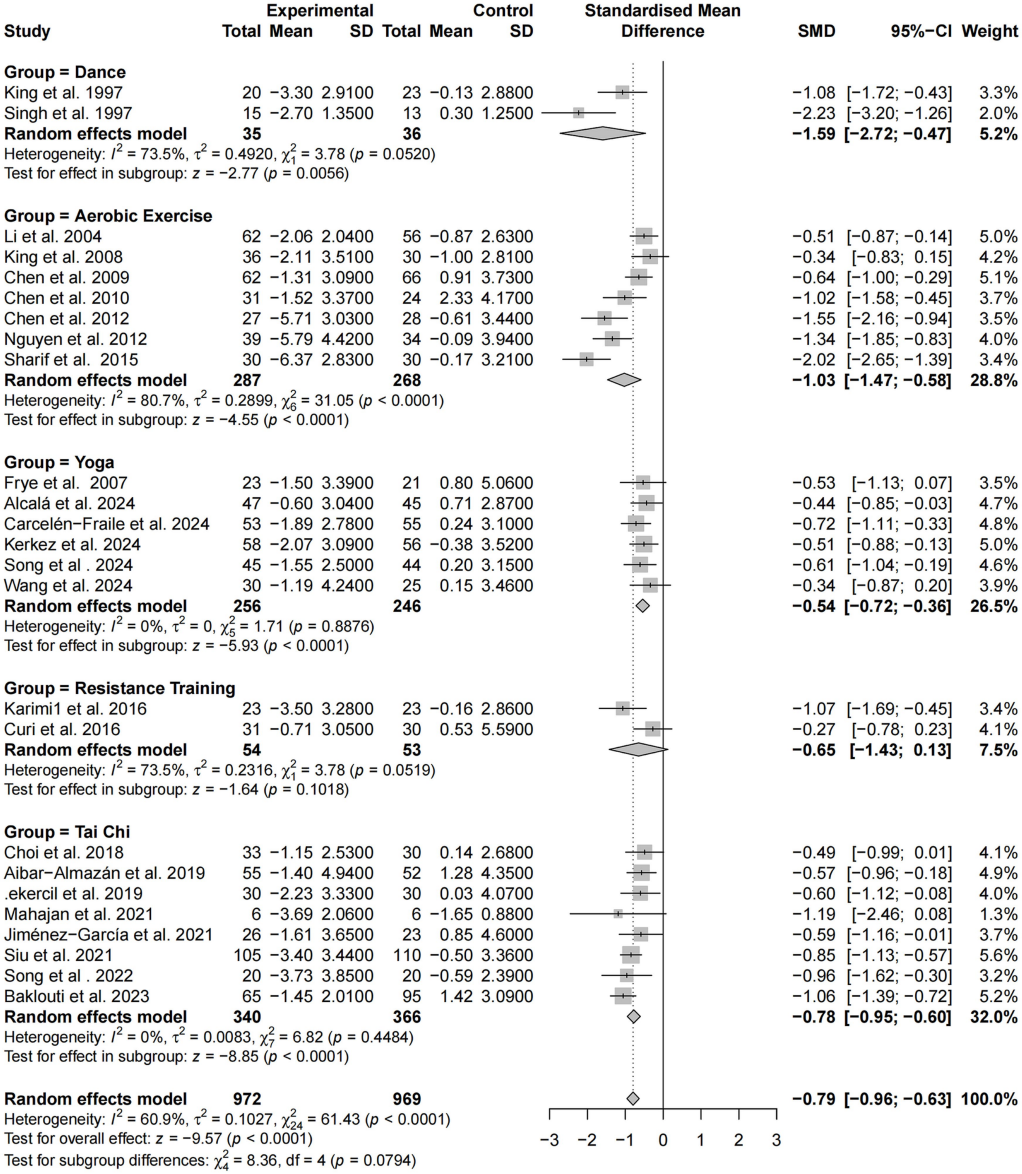
Subgroup analysis of intervention modality on sleep quality improvement.

Although the differences among subgroups approached statistical significance (*p =* 0.0794), the findings suggest that dance, aerobic exercise, and tai chi are particularly effective and replicable modalities for improving sleep quality, with dance-based interventions demonstrating the greatest potential benefit (Figure 10). It should be noted that SMD values were applied solely to the global PSQI score to harmonize differences in measurement scales across studies; no other sleep outcome measures were synthesized in this analysis.

### Meta-regression analysis

**Figure 11:**
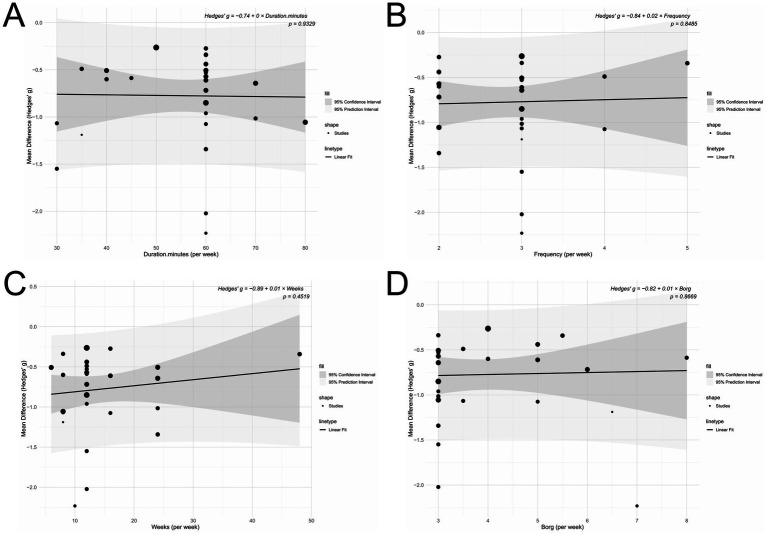
**(A)** Duration (minutes per week); **(B)** Frequency (per week); **(C)** Intervention period (weeks); **(D)** Intensity (Borg scale).

The meta-regression analysis revealed no statistically significant linear relationships between intervention effect and any of the following variables: session duration (*p* = 0.933), exercise intensity (*p =* 0.867), intervention frequency (*p =* 0.849), or total intervention duration (*p =* 0.452; Figure 11).

## Discussion

### Synthesis of evidence

Grounded in the 2024 Guidelines for Elderly Health, this study investigated the dose–response relationship between physical exercise and improvements in sleep among older adults using metabolic equivalents (METs). The results provide an evidence-based foundation for designing personalized sleep intervention prescriptions. The findings demonstrated that exercise interventions significantly improved subjective sleep quality in older adults. Among various intervention types, dance-based programs, aerobic exercise, and Tai Chi were particularly effective, demonstrating large and consistent improvements in subjective sleep quality. Although this study shows that the subgroup with low-intensity intervention had the greatest effect, it also indicates that moderate-intensity exercise yields significant benefits. This result is in line with the World Health Organization (WHO) guidelines of 2020 (43). Supporting literature indicates that 150–300 min of moderate-intensity exercise per week is sufficient to substantially enhance subjective sleep quality in the elderly (13).

The nonlinear dose–response model revealed an inverted U-shaped relationship, with the maximal intervention effect occurring at approximately 527 MET-min/week. Beyond this threshold, the benefit plateaued, indicating that moderate exercise doses are sufficient to produce substantial improvements in sleep quality.

Importantly, the findings further suggested that even lower exercise doses—approximately 300 MET-min/week—could yield significant improvements in subjective sleep quality, despite falling below the WHO’s recommendation of 600 MET-min/week. This implies that near-optimal effects can be achieved within a critical dosage range, minimizing unnecessary exercise burden. Such an approach is particularly well-suited for older adults with lower physical capacity or limited exercise tolerance. Zang et al. (15) also reported that just 300 MET-min/week of low-to-moderate intensity exercise could significantly alleviate frailty symptoms in older adults, with optimal benefits observed within the 600–1,200 MET-min/week range. This underscores that moderate exercise loads are both sustainable and adaptable, offering high safety and better long-term adherence among older populations—leading to consistent health outcomes (15).

Subgroup analyses revealed that intervention frequency and duration did not significantly influence the effectiveness of sleep improvement, suggesting these parameters may be less critical. However, trends were observed regarding exercise intensity and session duration: lower-intensity exercise and sessions lasting less than 30 min tended to produce more stable and sustainable improvements. These findings align with previous studies by Reid et al. (44), and Tseng et al. (45), which emphasized that moderate-intensity activities are more manageable for older adults and are associated with better compliance and outcomes. Reid et al. (44) demonstrated that moderate aerobic exercise improved insomnia symptoms in the elderly more effectively than high-intensity activity, which, due to the associated physiological demands (e.g., elevated heart rate, breathlessness), may reduce adherence.

It is particularly noteworthy that although high-intensity, long-duration exercise has shown potential benefits in some trials, older adults are more vulnerable to fatigue and fall-related injuries under such regimens. This has been explicitly noted by You et al. (46) and Crane et al. (47). Therefore, low-to-moderate intensity exercise with moderate session duration appears to be the most suitable approach for promoting sustained engagement and improving subjective sleep quality among older adults. Such interventions are not only safer but also better aligned with the physiological characteristics and health needs of this population.

Intervention strategies should prioritize safety while encouraging participation in enjoyable, gentle, and varied low-to-moderate intensity activities to enhance adherence and achieve long-term improvements in sleep and overall health. Flexibility in intervention design is particularly important for older adults, as it reduces fatigue, minimizes the risk of exercise-related injuries, and supports sustainable participation. Given the high prevalence of sleep disorders among elderly populations, rehabilitation specialists, exercise prescription developers, and researchers should develop personalized, moderate-intensity, and easy-to-follow programs—guided by the 2024 Guidelines for Elderly Health—to improve sleep quality and enhance overall well-being in a safe and adherent manner.

### Clinical implications of the dose–response analysis

This study highlights the critical role of exercise interventions in improving subjective sleep quality among older adults, demonstrating that even low-dose exercise programs can yield significant benefits. In clinical practice, many older individuals are unable to meet the World Health Organization’s recommended levels of physical activity due to physical limitations or mobility impairments. Against this backdrop, low-dose and low-intensity exercise interventions are especially important. Such approaches not only promote gradual adaptation to physical activity but also reduce the risk of excessive fatigue or falls. A progressive, stepwise intervention model can enhance physical function while ensuring safety, thereby improving both sleep quality and overall well-being in older adults (48).

Importantly, beyond statistical significance, the magnitude of improvement observed in the present meta-analysis also demonstrated clinical relevance. The weighted mean differences (WMD) for PSQI exceeded the minimal clinically important difference (MCID), which is generally defined as a reduction of approximately 3 points. This indicates that the improvements in sleep quality observed here are not only statistically robust but also meaningful from a clinical perspective, with tangible benefits for older adults experiencing sleep difficulties.

The findings further reinforce the importance of tailoring exercise prescriptions to individual needs. Intervention programs should be designed based on the elderly individual’s health status, preferences, and living environment (13). This person-centered approach supports the evolution of sleep improvement strategies toward greater precision and inclusivity across diverse populations (49).

Although evidence-based medicine has advanced significantly in the field of exercise and aging, substantial research gaps remain in understanding the mechanisms, optimal intensity thresholds, and intervention modalities for sleep disorders. Future studies are needed to explore the adaptability and differential effects of various exercise types, intensities, and frequencies across subpopulations with varying covariates. Dose–response analysis offers valuable insights for optimizing intervention strategies and enhancing their effectiveness, and this study seeks to provide a methodological reference for clinical practitioners and rehabilitation researchers in the field of sleep health.

### Strengths and limitations

This study systematically evaluated the relationship between exercise dose and improvements in subjective sleep quality among older adults, and proposed a theoretical framework for personalized exercise prescriptions. The research process adhered strictly to established meta-analysis guidelines, with comprehensive literature searches conducted across multiple authoritative databases to ensure scientific rigor and representativeness. Through standardized screening procedures and stringent quality control measures, the risk of selection bias was minimized, enhancing the credibility and robustness of the conclusions.

The findings offer a practical pathway for older adults with limited physical capacity and provide evidence-based support for developing more accessible intervention strategies for sleep disorders. Moreover, the study lays a theoretical foundation for future individualized and precision-oriented rehabilitation programs, with significant potential for clinical translation and public health impact.

Nevertheless, several limitations should be acknowledged. First, the inclusion of English-language publications only may introduce language bias and limit the global generalizability of the findings. Second, although subgroup analyses were conducted, the number of studies included in some subgroups was small, potentially reducing statistical power and result stability. Third, most of the included studies lacked long-term follow-up data post-intervention, restricting our ability to assess the sustainability and long-term efficacy of exercise interventions. Additionally, many original studies failed to report adherence to the exercise protocol and intervention fidelity, which may affect the accuracy of dose–response estimations.

Future research should aim to improve study quality and reporting completeness, extend follow-up durations to evaluate long-term effects, and incorporate covariate-adjusted subgroup analyses and mechanistic investigations. Such efforts are essential for strengthening the scientific basis and practical guidance of exercise interventions tailored to older populations.

## Conclusion

This study demonstrates that exercise interventions can significantly improve subjective sleep quality in older adults. The most pronounced effects were observed under conditions involving a frequency of 2–3 sessions per week, with each session lasting no more than 30 min and performed at low to moderate intensity. Notably, even low-dose interventions totaling approximately 300 MET-minutes per week were associated with substantial improvements, offering a practical and accessible pathway for older adults with limited physical capacity.

Given concerns related to safety and adherence, high-intensity or high-frequency exercise regimens are not recommended for this population. Future research should focus on elucidating the mechanisms underlying the long-term maintenance of intervention effects, and on optimizing exercise type, intensity, and frequency in an integrated manner. These efforts will be essential for developing more precise and sustainable exercise strategies tailored to the needs of older adults, and for advancing both theoretical and empirical support in the field of geriatric sleep health.

## Data Availability

The original contributions presented in the study are included in the article/Supplementary material, further inquiries can be directed to the corresponding author.
