# Supplementary material for: Effects of exercise interventions on subjective sleep quality in older adults: a systematic review and meta-analysis of studies using the Pittsburgh sleep quality index
Source: Front Med (Lausanne). 2025 Oct 2;12:1664567. doi: 10.3389/fmed.2025.1664567 (PMC12528139; doi:10.3389/fmed.2025.1664567)
Supplement: Supplementary file 1 [file Table_1.DOCX]

Section A

| **Detailed Search Strategies for Each Database** | |
| --- | --- |
| PubMed search: May 1, 2025 |  |
| ("sleep"[Title/Abstract] OR "sleep quality"[Title/Abstract] OR "slumber"[Title/Abstract] OR "rest"[Title/Abstract]) AND ("exercise"[Title/Abstract] OR "physical exercise"[Title/Abstract] OR "exercise training"[Title/Abstract] OR "physical activity"[Title/Abstract] OR "workout"[Title/Abstract]) AND ("older adults"[Title/Abstract] OR "elderly"[Title/Abstract] OR "aged adults"[Title/Abstract] OR "seniors"[Title/Abstract] OR "middle-aged and older adults"[Title/Abstract])) AND (randomizedcontrolledtrial[Filter]) | 265 |
| Cochrane Library search: May 1, 2025 |  |
| #1(sleep OR"sleep quality"OR slumber OR rest)  #2(exercise OR"physical exercise"OR"exercise training"OR"physical activity"OR workout)  #3(older adults OR"aged adults"OR seniors OR"middle-aged and older adults")  #4(randomized controlled trial) | 141 |
| Scopus search: May 1, 2025 |  |
| TITLE-ABS-KEY("sleep" OR "sleep quality" OR "slumber" OR "rest")  AND TITLE-ABS-KEY("exercise" OR "physical exercise" OR "exercise training" OR "physical activity" OR "workout")  AND TITLE-ABS-KEY("older adults" OR "elderly" OR "aged adults" OR "seniors" OR "middle-aged and older adults")  AND TITLE-ABS-KEY("randomized controlled trial") | 795 |
| Embase search: May 1, 2025 |  |
| (("sleep":ti,ab,kw OR"sleep quality":ti,ab,kw OR"slumber":ti,ab,kw OR"rest":ti,ab,kw)  AND("exercise":ti,ab,kw OR"physical exercise":ti,ab,kw OR"exercise training":ti,ab,kw OR"physical activity":ti,ab,kw OR"workout":ti,ab,kw)  AND("older adults":ti,ab,kw OR"aged adults":ti,ab,kw OR"seniors":ti,ab,kw OR"middle-aged and older adults":ti,ab,kw))  AND("randomized controlled trial") | 351 |
| Wed of secience search: May 1, 2025 |  |
| TS=("sleep" OR "sleep quality" OR "slumber" OR "rest")  AND TS=("exercise" OR "physical exercise" OR "exercise training" OR "physical activity" OR "workout")  AND TS=("older adults" OR "aged adults" OR "seniors" OR "middle-aged and older adults")  AND TS=("randomized controlled trial") | 347 |
